# Behavioral Risks for West Nile Virus Disease, Northern Colorado, 2003

**DOI:** 10.3201/eid1303.060941

**Published:** 2007-03

**Authors:** Indira B. Gujral, Emily C. Zielinski-Gutierrez, Adrienne LeBailly, Roger Nasci

**Affiliations:** * Larimer County Department of Health and Environment, Fort Collins, Colorado, USA; † Centers for Disease Control and Prevention, Fort Collins, Colorado, USA

**Keywords:** West Nile virus, prevention, behavior, DEET, insect repellent, research

## Abstract

In 2003, residents in 2 adjacent cities in northern Colorado (Loveland and Fort Collins) had severe outbreaks of human West Nile virus (WNV) disease. Unexpectedly, age-adjusted neuroinvasive disease rates were higher in Loveland (38.6 vs. 15.9 per 100,000), which had a more extensive mosquito control program and fewer mosquitoes. A survey was conducted to assess differences in personal protection and risk practices by each city's residents. During May and June 2004, a random-digit dial telephone survey was conducted among adults to assess personal protection behavioral practices used to prevent WNV infection during the 2003 outbreak. After we adjusted for identified risk factors, Loveland residents were 39% more likely to report seldom or never using N,N-diethyl-*m*-toluamide (DEET), and ≈30% were more likely to report being outdoors during prime mosquito-biting hours than Fort Collins residents. Personal protective practices may directly influence rates of WNV infection and remain important even when comprehensive community mosquito control measures are implemented.

## Abstract 2

Protective practices may affect the level of illness in a community.

In the United States, the mantra familiar to public health workers and residents living in West Nile virus (WNV)–affected areas is to practice the 4 Ds of prevention: 1) DEET (N,N-diethyl-*m*-toluamide): wear an insect repellent containing DEET; 2) dress: wear long sleeves and long pants; 3) drain: drain standing water around the home; and 4) dusk to dawn: limit time outdoors during this time. Although the 4 Ds have been used by many state and local health departments to promote personal prevention, the question remains, how well do these tactics work to prevent infection?

In 2003, ≈30% (2,947) of human WNV cases in the United States were reported in Colorado. Among infected residents, 63 died (*1*). WNV transmission was especially intense in northern Colorado, including Larimer County. Among county residents, 546 laboratory-confirmed cases of WNV disease, including 63 neuroinvasive disease cases and 9 deaths (*2*), occurred. Officials at the Larimer County Department of Health and Environment noted differences in age-adjusted rates of WNV neuroinvasive disease between the 2 largest cities in the county. WNV neuroinvasive disease rates were used because neuroinvasive cases are more likely to be captured in surveillance systems because of illness severity, which often requires hospitalization and prompt laboratory diagnosis, unlike the generally milder West Nile fever. Furthermore, WNV neuroinvasive disease cases are typically used to draw comparisons between geographic areas and assess rates over time (*3*,*4*). The city of Loveland had a much higher age-adjusted rate of neuroinvasive disease (38.6/100,000) than the city of Fort Collins (15.9/100,000); standardized risk ratio 2.43 (95% confidence interval [CI] 1.21–4.87, p<0.01). These findings were unexpected given the ecologic and demographic similarities of the 2 cities and a long-term comprehensive mosquito control program in Loveland.

Among the 265,489 Larimer County residents in July 2003, most lived in the cities of Fort Collins (125,461) and Loveland (55,905) (*5*). Although some limited ecologic differences exist (i.e., Loveland water surface area is ≈2.5× greater), Fort Collins and Loveland are largely similar. Demographically, both cities are ≈90% white with 9% reported Hispanic ethnicity, 8%–13% of the residents are ≥65 years of age, and the annual household estimated median income is ≈$45,000 per year (*5*).

Loveland and Fort Collins are both situated in a high plains ecologic zone. The dominant WNV vector mosquito species are *Culex tarsalis* and *Cx*. *pipiens* (*6*). During the 2003 outbreak, ≈20–40 mosquito traps were collected per city per week by using CO_2_ baited Centers for Disease Control and Prevention (CDC) miniature light traps. Mosquitoes were collected by the Colorado Mosquito Control (Brighton, CO, USA) and the Division of Vector-Borne Infectious Diseases at CDC (Fort Collins, CO, USA). During the height of the outbreak, from July 26, 2003, to September 5, 2003, the mean ± standard deviation number of *Cx*. *tarsalis* and *Cx*. *pipiens* mosquitoes collected per trap night was higher in Fort Collins (*Cx* *tarsalis* 76 ± 62, *Cx*. *pipiens* 31.5 ± 13.2,) than in Loveland (*Cx*. *tarsalis* 43 ± SD 34, *Cx*. *pipiens* 7 ± 1) (Figure). On the basis of 7,037 mosquitoes tested (4,999 *Cx*. *tarsalis* and 2,038 *Cx*. *pipiens*), the WNV infection rates (estimated number of mosquitoes infected/1,000 tested) were approximately equivalent in the 2 cities during that period (*Cx* *tarsalis* 14.7 in Fort Collins, 12.8 in Loveland; *Cx*. *pipiens* 25.9 in Fort Collins, 21.2 in Loveland). A vector index was calculated to estimate the average number of WNV-infected mosquitoes collected per trap night (i.e., summation of the product of the average number *Culex* mosquitoes collected per trap night and the proportion infected for each species). More WNV-infected mosquitoes were present in Fort Collins than in Loveland (Figure ) (CDC, unpub. data). This finding was consistent with mosquito control efforts occurring during that period; Loveland had an integrated mosquito control program in place since 1986, and Fort Collins reacted to the outbreak by implementing an emergency mosquito control program later in the outbreak (mid-August through early September).

**Figure:**
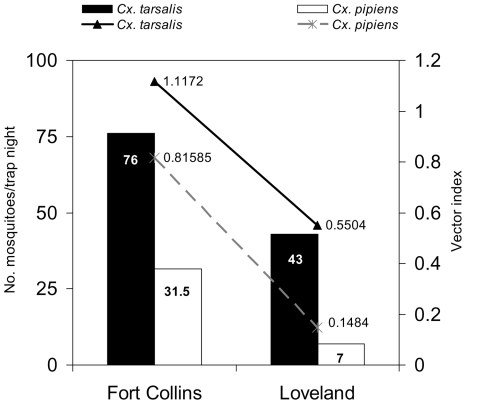
*Culex tarsalis* and *Cx. pipiens* density (average number collected per trap night) and vector index (reflecting the average number of infected mosquitoes collected per trap night), Fort Collins and Loveland, Colorado, July 26, 2003 through September 5, 2003.

Larimer County health officials encouraged residents to “Fight the Bite,” specifically, to practice the 4 Ds of prevention. The difference in age-adjusted rates of WNV neuroinvasive disease between the 2 cities was unexpected because Fort Collins residents were exposed to a larger number of WNV-infected mosquitoes. To understand the differences in rates of neuroinvasive disease in the 2 cities, Larimer County health officials commissioned a survey to assess city residents’ knowledge, attitudes, and beliefs about WNV and to measure reported personal protective practices during the 2003 WNV season. The purpose of this study was to increase our understanding of the role of individual preventive measures by assessing post hoc the behavioral practices among residents of Fort Collins and Loveland.

## Methods

From May 4 to June 7, 2004, the Survey Research Unit of the Colorado Department of Public Health and Environment in Denver performed a random-digit-dial telephone survey among residents of Fort Collins and Loveland. The survey was developed by the Larimer County Department of Health and Environment, CDC, and the Colorado Department of Public Health and Environment. The survey consisted of 42 questions; 27 (64.0%) questions were specifically related to WNV, and 15 (36.0%) questions concerned demographic information. Questions were derived from the 2003 Colorado Behavioral Risk Factor Surveillance System, local agency staff, and a survey conducted by the Mississippi Department of Health (*7*,*8*).

All households with telephones in Fort Collins and Loveland were eligible for inclusion. One adult ≥18 years of age who lived within the city limits of Fort Collins or Loveland from July through August 2003 was randomly selected from each household to participate. Each phone number in the sample was called ≤15 times, with at least 3 attempts in the evening, 3 during the day, and 3 on the weekend until the total number of desired completed interviews was obtained. Interviews were conducted in either English or Spanish. All interviews were completed by using Computer-Assisted Telephone Interviewing software (Sawtooth Technologies, Northbrook, IL, USA).

The exposure factor of interest was residence (Fort Collins vs. Loveland). Outcomes were based on self-reported WNV preventive practices during the 2003 outbreak. Five outcomes were used: DEET repellent use (DEET was the only insect repellent active ingredient recommended in 2003); draining standing water; dressing in long clothing (pants and long-sleeved shirts); minimizing hours outside from dusk to dawn on weekends; and minimizing hours outside from dusk to dawn on weekdays. Outcome variables were dichotomized as follows: DEET and dress (sometimes, nearly always, or always vs. seldom or never); drain (yes vs. no); dusk to dawn on weekends (≤2 h vs. 3–40 h outside); and dusk to dawn on weekdays (≤5 h vs. 6–60 h outside).

Explanatory variables investigated included sex, age, ethnicity, income, education, owning an air-conditioner or swamp cooler, WNV risk perception, and awareness of a local mosquito control program. Variables were classified as follows: sex (male and female); age in years (18–44 and ≥45); ethnicity (non-Hispanic and Hispanic); income in US dollars (<$25,000, $25,000–50,000, and >$50,000); education (a high school diploma or less and college or higher); an air-conditioner or swamp cooler in home (yes and no); risk perception (very worried, somewhat worried, and not worried about getting sick from WNV); and awareness of a mosquito control program in city of residence (yes vs. no).

Analysis was conducted by using anonymized data. A Z test was used to identify statistically significant (p<0.05) differences in the proportion of residents reporting selected characteristics. Five unconditional logistic regression models were used to estimate the odds of each outcome among Fort Collins and Loveland residents while adjusting for identified risk factors. Models were built by using purposeful selection, a 5-step method for selecting variables based on both biologic importance and statistical significance. As outlined by Hosmer and Lemeshow (*9*), the 5 steps of purposeful selection are 1) test for univariate significance (p<0.25); 2) build the multivariate model (p<0.05); 3) test for confounding; 4) assess continuous variables for linearity; and, 5) test for effect modification and include interaction terms that are both significant (p<0.05) and biologically plausible. Model fit was determined by using the Hosmer–Lemeshow goodness-of-fit test (*9*). Because 5-point Likert scales can place persons in the middle category (*10*), final unconditional logistic regression models were retested to determine if similar results would be found between models in which the response “sometimes” was included in the opposite category (seldom and never). Statistical analyses were performed by using SAS version 9.1 software (SAS, Cary, NC, USA).

## Results

Among the 3,739 county households identified, 1,230 were sampled. Of these, 256 (20%) refused sampling 11 (1.1%) terminated the interview before it was completed, 6 (0.9%) had a language barrier, and 957 (78%) were surveyed. Fort Collins residents included 424 (44.3%) of those interviewed. Survey demographics reflect the general populations of Loveland and Fort Collins (*5*).

Among those surveyed, significant (p<0.05) differences were found between Fort Collins and Loveland residents by sex, age group, income, education, and ownership of an air-conditioner or swamp cooler. Compared with Loveland survey participants, more residents of Fort Collins were female, younger (18–44 years of age), had a higher income (>$50,000), educated (more than a high school diploma), and reported no air-conditioner or swamp cooler in the home (Table 1).

**Table 1:** Distribution of Fort Collins and Loveland residents by selected characteristic (potential risk factor), Larimer County West Nile Virus survey, Colorado, 2003

Characteristic	Fort Collins (n = 424) 44.3%, no. (%)	Loveland (n = 533) 55.7%, no. (%)	Total (N = 957) 100.0%, no. (%)
Sex			
Male	166 (39.1)	249 (46.7)	415 (43.4)*
Female	258 (60.9)	284 (53.3)	542 (56.6)*
Age group, y			
18–44	212 (50.0)	204 (38.3)	416 (43.5)*
≥45	212 (50.0)	325 (60.9)	537 (56.1)*
Unknown	0	4 (0.8)	4 (0.4)
Ethnicity			
Non-Hispanic	392 (92.5)	498 (93.4)	890 (93.0)
Hispanic	32 (7.5)	30 (5.7)	62 (6.4)
Unknown	0	5 (0.9)	5 (0.6)
Income, US $			
<25,000	72 (16.9)	106 (19.8)	178 (18.7)
25,000–50,000	121 (28.8)	159 (29.8)	280 (29.2)
>50,000	195 (45.9)	207 (38.8)	402 (42.0)*
Unknown	36 (8.4)	61 (11.6)	97 (10.1)
Education			
High school diploma or less	89 (21.0)	174 (32.8)	263 (27.5)*
College†	332 (78.3)	356 (66.7)	688 (71.9)*
Unknown	3 (0.7)	3 (0.5)	6 (0.6)
Air-conditioner, swamp cooler, or both, in home			
Yes	296 (69.8)	420 (78.8)	716 (74.8)*
No	128 (30.2)	113 (21.2)	241 (25.2)*
Risk perception			
Very worried	60 (14.1)	68 (12.8)	128 (13.4)
Somewhat worried	229 (54.0)	287 (53.8)	516 (53.9)
Not worried	134 (31.6)	177 (33.2)	311 (33.0)
Unknown	1 (0.2)	1 (0.2)	2 (0.2)
Knowledge of mosquito control program			
Yes	332 (78.3)	408 (76.5)	740 (77.3)
No and did not know	92 (21.7)	125 (23.5)	217 (22.7)

*Statistically significant differences by Z test (p<0.05) †College: some college, college degree, some graduate school, or graduate degree.

Significant differences (p<0.05) between Fort Collins and Loveland residents were observed for those reporting DEET use and those spending time outdoors from dusk to dawn on both weekends and weekdays (Table 2). The proportion of persons who reported seldom or never using DEET was higher among Loveland residents than among Fort Collins residents. Likewise, a higher proportion of Loveland residents reported spending >2 h outdoors from dusk to dawn on weekends and spending >5 h outdoors from dusk to dawn on weekdays.

**Table 2:** Distribution of Fort Collins and Loveland residents by outcome, Larimer County West Nile Virus survey, Colorado, 2003

Outcome*	Fort Collins (n = 424) 44.3%, no (%)	Loveland (n = 533) 55.7%, no. (%)	Total (N = 957) 100.0%, no. (%)
DEET use			
Sometimes/nearly always/always	271 (63.9)	298 (55.9)	569 (59.5)†
Seldom/never	147 (34.7)	223 (41.8)	370 (38.6)†
Unknown	1 (1.4)	12 (2.3)	18 (1.9)
Drain water			
Yes	192 (45.3)	213 (39.9)	405 (42.3)
No	67 (15.8)	92 (17.2)	159 (16.6)
Not applicable	165 (38.9)	227 (42.8)	393 (41.0)
Unknown	0 (0.0)	1 (0.1)	1 (0.1)
Dress (long sleeves and pants)			
Sometimes/nearly always/always	100 (23.6)	141 (26.5)	241 (25.2)
Seldom/never	134 (31.6)	186 (34.9)	320 (33.4)
Unknown	190 (44.8)	206 (38.6)	396 (41.4)
Dusk to dawn, weekends			
≤2 h outside	191 (45.0)	200 (37.5)	391 (40.8)†
3–40 h outside	174 (41.0)	234 (44.0)	408 (42.6)
Unknown	59 (14.0)	99 (18.5)	158 (16.6)
Dusk to dawn, weekdays			
≤5 h outside	261 (61.6)	279 (52.4)	540 (56.4)†
6–60 h outside	136 (32.0)	202 (38.0)	338 (35.3)
Unknown	27 (6.4)	52 (9.6)	79 (8.3)

*DEET, N,N-diethyl-*m*-toluamide.  †Statistically significant differences by Z test (p<0.05).

Five unconditional multivariate logistic regression models were built to test for an association between city of residence and reported WNV preventive behavior. The drain model was omitted after careful review of the survey question deemed it too vague for a meaningful interpretation. This was unfortunate because draining water from around a residence may reduce exposure to mosquito-breeding sites.

When we adjusted for sex, age, and risk perception, Loveland residents were 39% (95% confidence interval [CI] 1.04–1.76) more likely to report that they seldom or never used DEET than Fort Collins residents (Table 3). Among residents surveyed about DEET use, persons >45 years of age were 62% (95% CI 1.21–2.18) more likely to report seldom or never using DEET than younger respondents. Persons who were not worried about WNV were 4× (95% CI 2.90–7.51) more likely to report that they seldom or never used DEET than persons who sometimes, nearly always, or always worried about WNV. Similar results were obtained when the model was tested with the sometimes response included in the seldom or never group.

**Table 3:** Adjusted odds ratios and 95% confidence intervals for logistic regression models* assessing self-reported West Nile virus preventative outcomes by identified risk factors, Larimer County West Nile Virus survey, Colorado, 2003

Risk factors	DEET (n = 842), seldom/never use DEET	Dress (n = 507), seldom/never wear long clothes	Dusk to dawn, weekdays (n = 797), >5 h outside from dusk to dawn	Dusk to dawn, weekends (n = 795), >2 h outside from dusk to dawn
City				
Fort Collins	Referent	Referent	Referent	Referent
Loveland	1.39 (1.04–1.76)	1.02 (0.70–1.48)	1.35 (1.01–1.82)	1.30 (1.00–1.74)
Sex				
Female	Referent	Referent	Referent	Referent
Male	0.92 (0.69–1.25)	1.36 (0.94–1.98)	0.97 (0.63–1.48)	1.20 (0.90–1.61)
Age, y				
18–44	Referent	Referent	Referent	Referent
≥45	1.62 (1.21–2.18)	0.70 (0.48–1.01)	0.91 (0.67–1.22)	0.67 (0.51–0.89)
Education				
College†		Referent	Referent	
High school diploma or less		0.89 (0.58–1.38)	1.41 (0.99–1.98)	
Income, US $				
>50,000		Referent	Referent	
<25,000		0.79 (0.47–1.31)	0.65 (0.38–1.11)	
25,000–50,000		0.72 (0.47–1.08)	1.09 (0.69–1.72)	
Risk perception				
Very worried	Referent	Referent		Referent
Somewhat worried	1.47 (0.92–2.36)	1.79 (0.88–3.61)		1.68 (1.07–2.65)
Not worried	4.68 (2.90–7.51)	2.58 (1.25–5.28)		2.14 (1.31–3.51)
Interaction			Referent	
Male <$25,000			4.36 (1.88–10.0)	
Male $25,000–50,000			1.39 (0.72–2.69)	

*Hosmer-Lemeshow Goodness-of-fit is based on deciles of risk. p values >0.05 indicate good fit (N,N-diethyl-*m*-toluamide [DEET], p = 0.67; dress, p = 0.68; dusk/dawn, weekdays, p = 0.97; dusk/dawn, weekends, p = 0.91). †College: some college, college degree, some graduate school, or graduate degree.

After we adjusted for identified risk factors, no statistically significant difference was observed between Fort Collins and Loveland residents who reported seldom or never wearing long clothes to protect against mosquitoes. However, persons who reported that they were not worried about getting sick from WNV were 2.5× (95% CI 1.25–5.28) more likely to report not wearing protective clothing. Similar results were obtained when the sometimes response was included in the seldom or never response group.

Participants were surveyed regarding amount of time spent outdoors from dusk to dawn during the week and on weekends. Compared with the Fort Collins residents, Loveland survey participants were 35% (95% CI 1.01–1.82) more likely to report spending >5 h outdoors during the week from dusk to dawn when adjustments were made for sex, age, education, and income. The model also held a statistically significant and plausible interaction term; males earning <$25,000 per year were more likely to report spending > 5 h outside from dusk to dawn during the week.

Compared with Fort Collins residents, Loveland residents were 30% (95% CI 1.00–1.74) more likely to report spending >2 h outdoors from dusk to dawn on weekends when adjustments were made for sex, age, and risk perception. Similarly, persons who reported they were somewhat worried or not worried about getting sick from WNV were 68% and >2× as likely (95% CI 1.07–2.65 and 1.31–3.51), respectively, to report spending >2 h outdoors from dusk to dawn on weekends than persons very worried about getting sick from WNV.

## Discussion

During the 1999 WNV outbreak on Staten Island, New York, a serosurvey conducted by Mostashari et al. found the highest seroprevalence of WNV among persons who spent >2 h outdoors from dusk to dawn; persons were even more likely to be seropositive if they reported never using a repellent containing DEET (*11*). In a recent national study, 40% of survey participants reported using a repellent containing DEET and draining standing water, 29% avoided perceived areas with mosquitoes, 28% avoided being outdoors from dusk to dawn, and 27% wore long sleeved-shirts and long pants to avoid bites (*12*).

The results of this study suggest that differences in WNV neuroinvasive disease rates may be due, in part, to lower use of repellents containing DEET and greater dusk-to-dawn outdoor exposure among Loveland residents. These findings support the benefit of promoting personal prevention approaches, particularly by using effective insect repellents and reducing exposure to mosquitoes during prime-biting hours.

An alternative explanation for the differences in neuroinvasive disease rates among Loveland and Fort Collins residents may be unexplained ecologic differences that influence the risk for infection. Loveland has a greater proportion of water surface area than Fort Collins, a difference that has been hypothesized to influence mosquito populations, local bird populations, and human behavior. However, on the basis of vector indices for 6 weeks of entomologic data collected during the height of the 2003 outbreak, more WNV-infected mosquitoes were present in Fort Collins than in Loveland. This finding was predictable, given that Fort Collins implemented an emergency mosquito control program late in the WNV season.

Many results of this study are consistent with those of previous reports. Older persons and those not worried about WNV infection were more likely to report seldom or never using a repellent containing DEET (*12*,*13*). Similarly, persons with lower incomes reported practicing fewer preventive behavioral measures. This finding was evident in the dusk-to-dawn weekday model in which an interaction term appeared; men with the lowest income levels were more likely to be outdoors during the week from dusk to dawn. This result seems plausible given that weekday workers have less control over outdoor exposure than nonworking hours during the weekend and may specifically capture those engaged in agricultural or landscaping work. No differences between Fort Collins and Loveland residents were observed for those reporting seldom or never wearing long clothes, which is not surprising, given that few people use this strategy (*12*).

Although explanatory variables help identify the proportion of surveyed persons not following the 4 Ds of prevention, they do not explain why Loveland residents were less likely to practice personal prevention behavioral measures. Loveland residents may have had less knowledge of these prevention strategies. However, this was unlikely, given widespread WNV educational efforts in both cities and local and state media coverage of the outbreak. Furthermore, bivariate analysis of reported risk perception indicates that both Fort Collins and Loveland residents perceived very similar risks for WNV infection.

Perceived risk for disease was a consistent factor in the multivariate models. Persons who were not worried about WNV were more likely to report seldom or never using a repellent with DEET, not wearing long clothes, and spending more time outdoors from dusk to dawn on the weekend. As noted by other authors (*14*–*16*), risk perception is only one of many factors that directly contribute to practicing preventive behavioral measures. For example, environmental triggers may play a role. In a model proposed by Zielinski-Gutierrez and Hayden, a person’s experience with their environment (i.e., seeing mosquitoes, getting bitten, or both) is one of the most immediate triggers for taking protective action (*17*). This was true for residents in Mississippi who in 2003 reported feeling a mosquito bite as the most important reason for taking precautions against mosquito bites (*8*). During the outbreak in Larimer County, biting pressure from the nuisance mosquito *Aedes vexans* (Fort Collins, 39.6/trap night), and Loveland (22.6/trap night) along with *Culex* sp. may have prompted residents to use repellent and practice other avoidance strategies. Environmental triggers, such as biting pressure, may explain why Fort Collins and Loveland residents responded similarly to a general question on risk perception regarding WNV but reported differences in preventive behaviors.

This possibility raises a related question, “Did Loveland residents choose to rely on the city’s control program instead of practicing individual preventive measures?” Loveland residents may have been less likely to have applied personal preventive measures (the 4 Ds of prevention) given their reliance on the long-standing community mosquito control program. Although difficult to establish with any certainty, this prospect suggests the need to promote integrated prevention with both community and individual actions complementing each another. Future research should assess the multiple factors that contribute to risk perception and address the human-environmental interactions that influence protective behaviors.

Although this study is limited by recall and reporting bias because the survey was conducted 8 months after the outbreak, these information biases are most likely nondifferential since recall and reporting would likely be similar among both Loveland and Fort Collins residents. The results of this study reinforce use of personal protection efforts even in areas with strong community mosquito control measures and suggest that these personal measures may influence disease rates. Furthermore, study results suggest that persons residing in a city with greater mosquito-biting pressure, as measured by a vector index, were more likely to take preventive measures than persons in a community with less biting pressure. Future studies are warranted to understand the effects of human-environment interactions to derive the greatest benefit from community and personal efforts to reduce disease and death from WNV.

## References

[1] Centers for Disease Control and Prevention. West Nile virus statistics, surveillance, and control. Atlanta: The Centers; 2006.

[2] Colorado Department of Public Health and Environment. Human West Nile virus infections: Colorado, 2005 [cited 2006 Dec 26]. Available from http://www.cdphe.state.co.us/dc/zoonosis/wnv/HUMAN_WNV_03.HTML.

[3] Centers for Disease Control and Prevention. Epidemic/epizootic West Nile virus in the United States: guidelines for surveillance, prevention, and control. Atlanta: The Centers; 2003. p. 18–20.

[4] Centers for Disease Control and Prevention. West Nile virus activity—United States, January 1–December 1, 2005. MMWR Morb Mortal Wkly Rep. 2005;54:1253–6. 16357821

[5] United States Census Bureau. State and county quickfacts. Fort Collins (CO): United States Census Bureau; 2005.

[6] Hayes EB, Komar N, Nasci RS, Montgomery SP, O’Leary DR, Campbell GL. Epidemiology and transmission dynamics of West Nile virus disease. Emerg Infect Dis. 2005;11:1167–73. 16102302 10.3201/eid1108.050289a PMC3320478

[7] Mississippi Department of Health. West Nile virus. Jackson (MS): The Department; 2006.

[8] Slavinski S, Jones E. Evaluation of the Mississippi fight the bite campaign. CDC Fifth National Conference on West Nile Virus in the United States. Feb 3–5, 2004. Atlanta: Centers for Disease Control and Prevention; 2004.

[9] Hosmer DW, Lemeshow S. Applied logistic regression. 2nd ed. New York: Wiley-Interscience; 2000.

[10] Benson PH. How many scale categories shall we use in consumer research? J Mark. 1970;35:59–61. 10.2307/1250460

[11] Mostashari F, Bunning ML, Kitsutani PT, Singer DA, Nash D, Cooper MJ, Epidemic West Nile encephalitis, New York, 1999: results of a household-based seroepidemiological survey. Lancet. 2001;358:261–4. 10.1016/S0140-6736(01)05480-0 11498211

[12] Blendon R. Residents of states reporting most West Nile virus cases are less likely to take precautions against mosquitoes. Press Release. Boston: Harvard School of Public Health; 2004.

[13] McCarthy TA, Hadler JL, Julian K, Walsh SJ, Biggerstaff BJ, Hinten SR, West Nile virus serosurvey and assessment of personal prevention efforts in an area with intense epizootic activity: Connecticut, 2000. Ann N Y Acad Sci. 2001;951:307–16. 11797787 10.1111/j.1749-6632.2001.tb02706.x

[14] Conner M, Norman P. Predicting health behaviour: research and practice with social cognition models. Philadelphia: Open University Press; 1996.

[15] Weinstein ND. Testing four competing theories of health-protective behavior. Health Psychol. 1993;12:324–33. 10.1037/0278-6133.12.4.324 8404807

[16] Brewer NT, Weinstein ND, Cuite CL, Herrington JE. Risk perceptions and their relation to risk behavior. Ann Behav Med. 2004;27:125–30. 10.1207/s15324796abm2702_7 15026296

[17] Zielinski-Gutierrez EC, Hayden MH. A model for defining West Nile virus risk perception based on ecology and proximity. EcoHealth. 2006;3:28–34. 10.1007/s10393-005-0001-9

